# Dignity encounters: the experiences of people with long-term illnesses and their close relatives within a primary healthcare setting

**DOI:** 10.1017/S1463423622000603

**Published:** 2022-11-14

**Authors:** Anna Nygren Zotterman, Lisa Skär, Siv Söderberg

**Affiliations:** 1 Department of Health Science, Luleå University of Technology, Luleå, Sweden; 2 Department of Health, Blekinge Institute of Technology, Karlskrona, Sweden; 3 Department of Nursing Sciences, Mid Sweden University, Sundsvall, Sweden

**Keywords:** accessibility, close relatives, confirmation, dignity encounter, interviews, people with long-term illnesses, primary healthcare, thematic content analysis

## Abstract

**Aim:**

To describe the experiences of dignity encounters from the perspective of people with long-term illness and their close relatives within a primary healthcare setting.

**Background:**

The importance of dignity as a concept in nursing care is well known, and in every healthcare encounter, the patient’s dignity has to be protected.

**Methods:**

A purposive sample of 10 people (5 couples) participated in this qualitative descripted study. One person in each of the couples had a long-term illness. Conjoint interviews were conducted and analyzed with an inductive qualitative content analysis.

**Results:**

The analysis resulted in three themes: i) *Being supported by an encouraging contact*; ii) *Being listen to and understood*; and iii) *Being met with respect*. Couples described being encountered with dignity as having accessibility to care in terms of being welcomed with their needs and receiving help. Accessibility promoted beneficial contact with healthcare personnel, who empowered the couples with guidance and support. Couples described a dignity encounter when healthcare personnel confirmed them as valuable and important persons. A dignity encounter was promoted their sense of feeling satisfied with the care they received and promoted safe care. Treated with dignity had a positive impact on the couples’ health and well-being and enhanced their sense of a good impression of the healthcare personnel within the primary health care.

**Conclusions:**

Healthcare personnel must regard and consider people with long-term illnesses and their close relatives’ experiences of dignity encounters to gain an understanding that enables them to support their needs and to know that the care is directed toward them.

## Introduction

In every healthcare encounter, each patient’s dignity has to be protected (Hoffman, 2002). Every human interaction has the potential to be a dignity encounter based on collaboration in which the person’s dignity is either promoted or violated (Jacobson, 2009). Modern healthcare described as moving away from a paternalistic system to a person-centered approach in which the healthcare personnel aim to work in partnership with the patient, promoting his or her autonomy and dignity (Walsh and Kowanko, 2002). Despite this, research has shown that there exists dignity violation in healthcare today through encounters characterized by rudeness, disregard, objectification, discrimination, assault, and abjection (Söderberg, 1999, Söderberg *et al*., 1999; Jacobson, 2009; Juuso *et al*., 2014; Skär and Söderberg, 2018). A condition that usually promotes this is characterized by an asymmetrical relationship between the actors in the healthcare setting and can be caused by a number of tensions (Skär and Söderberg, 2012; Söderberg *et al*., 2012; Skär and Söderberg, 2018). According to Baillie and Gallagher (2011), preserving patient dignity is the essence for effective relationships between healthcare professionals and patient.

Dignity characterizes the substance of nursing care, and nurses have a professional responsibility to promote and preserve every patient’s dignity. Human dignity is about respecting oneself in addition to respecting others (Manookian *et al*., 2014). Nordenfelt (2004) described dignity as a concept related to the right to respect and self-respect. The dignity of a person is worthy of respect from others and from the person himself or herself and belongs to the subject. According to Milton (2008), the concept of dignity refers to the quality of being worthy, honored, or esteemed grounded in various definitions of a human attribute or human rights in life. Edlund *et al*. (2013) have studied the phenomenon of dignity in several studies; this particular study aims to gain a determination of dignity as a concept. Dignity is based on a source of values described as absolute and relative dignity. The values connected to absolute dignity are holiness, human worth, freedom, responsibility, duty, and serving one’s fellow humans, and these values are absolute and impossible to deny every human being. Relative dignity is a reflection of absolute dignity and consists of a source of values; it is influenced and shaped by culture and society, which allow it to be altered, destroyed, and restored (Milton, 2008). These relative values can be violated and thus lead to the experience of a loss of dignity.

Patients have described that their dignity was maintained not only when they were seen as persons, meaning that their personalities had been acknowledged and respected, but also when they had control of their care (Walsh and Kowanko, 2002). Studies (Schröder *et al*., 2007; Matiti and Trorey, 2008; Baillie, 2009; Jonasson *et al*., 2010) showed that patients and close relatives valued being treated with respect in their relationship with healthcare personnel; being listened to, welcomed, and seen as worthy were mentioned as part of being met with dignity. Jonasson *et al*. (2010) stated that, when respect is present, it can be seen as a caring encounter that rests upon ethical values. Patient dignity is further related to a feeling of not only being comfortable and being safe and to a sense of well-being but also being valued in the healthcare relationship (Baillie, 2009). According to Schröder *et al*. (2007), good relations and communication based on dignity, security, and participation among healthcare personnel, patients, and close relatives can be seen as central and important factors regarding the quality of care in health care. Dignity encounters that result in a violation more likely to appear when one person in the relationship is in a position of vulnerability, for example, being affected by an illness, and the other person is in a position of antipathy, meaning that the person is arrogant, hostile, or impatient (Jacobson, 2009). It is more common for dignity to be promoted when the relationship is based on confidence and compassion. This means that the relationship is characterized by qualities such as empathy and trust, and that the people in the relationship have good intentions toward each other.

Within the field of primary healthcare (PHC), it is important that nurses view the close relatives as an integrated part of the patient’s life, with needs and experiences, and that they are included in the nursing care of the patient as the close relative is an everyday aspect of most people’s lives (Fast Braun and Foster, 2011). Close relatives’ presence and support are important, both for the ill person and for the healthcare personnel (Engström and Söderberg, 2007). Although the literature on dignity is extensive, research from the perspective of people with long-term illness and their close relatives’ experiences of dignity encounters within a PHC setting remains scarce. Understanding dignity in healthcare encounters can be important and helpful when it comes to taking note of the need for the person who is ill and the close relatives as companions when visiting the PHC. Being treated with dignity by healthcare personnel can support patients’ health and well-being and create the conditions for a respectful environment in the PHC setting. Experiences of being treated with dignity should always have a central place within the caring sciences, that is, nursing care (Eriksson, 1994; Edlund, 2002). Dignity is a prerequisite for a caring and trusting relationship where the patients and close relatives’ needs and resources are supported and protected. Research (Söderberg, 1999; Edlund, 2002) has shown that when a caring relationship is based on respect, kindness, and dignity it is caring. This study can contribute to a deeper understanding and can influence the quality of care in meetings with healthcare personnel. Thus, the aim of this study was to describe the experiences of dignity encounters from the perspective of people with long-term illness and their close relatives within a PHC setting.

## Methods

### Study design

A qualitative descriptive design was used in this study to describe people with long-term illness and their close relatives’ experiences of dignity encounters within a PHC setting. Data were collected through conjoint interviews (cf. Torgé, 2013) and analyzed using inductive qualitative content analysis (cf. Downe-Wamboldt, 1992).

### Participants and procedure

A purposive sample of five couples (five women and five men) participated in this study. The data collection was performed with joint interviews to capture the couples shared experience of dignity in encounters in health care. This means that participants who could provide information, knowledge, and a willingness to share their experiences were selected. The total of 10 participants were between 65 and 87 years of age (md = 73). The inclusion criteria were that they were couples and that one person in the couple was diagnosed with a long-term illness, and that both persons were willing to talk about their experiences of dignity in encounters with healthcare personnel in the PHC setting. In this study, the term healthcare personnel refer to general practitioners (GPs), nurses, district nurses, physiotherapists, and occupational therapists.

Of the five couples, three were married and two were cohabiting. The person in each couple who had been diagnosed with a long-term illness had multiple and different diagnoses, such as cerebral hemorrhage, arthritis, rheumatoid arthritis, chronic kidney disease, or diabetes. To facilitate recruitment, the first author contacted a patient association in the northern part of Sweden to ask whether the chairperson could give information about the study to potential participants and enquire whether they were interested in participation. The participants received both verbal and written information about the study aim during the meeting, and the couples who were interested in participating signed a list with their contact information in order to receive further contact. The first author contacted the couples and arranged an appointment to conduct the interviews.

### Interviews

Conjoint interviews (Torgé, 2013) were conducted with the participants. A pre-existing relationship is a premise in this type of dyadic interview. A condition to be called a dyad is the experience of a “we relationship,” formed through shared time and space together (Torgé, 2013). The conjoint interview is when the dyad is interview simultaneously. In this study, the five dyads are couples who share daily life since years ago. An interview guide was used, and the opening question was: Please, describe a dignity encounter in a meeting with healthcare personnel when visiting the healthcare center. The subsequent questions were Please, describe a dignity encounter you have experienced with the healthcare personnel? Please, give examples? Please, describe an incident when your dignity was affected? Please, describe what made an encounter dignified or not dignified? Please, describe what healthcare personnel should do to promote a dignity encounter in the PHC setting? Please, describe how a dignity encounter should be designed? Clarifying questions such as the following were asked: Can you please give an example? What do you mean by that? The first author conducted the interviews with the couples in their homes. Each interview lasted between 48 and 67 min (md = 57) was digitally recorded and then transcribed verbatim by the first author.

### Data analysis

Data were analyzed using inductive qualitative content analysis described by Downe-Wamboldt (1992). The analysis started by reading all the interview texts several times to achieve a sense of the content. This step was followed by a reading to identify meaning units that corresponded to the aim of the study. The meaning units were then condensed and coded. The codes were compared based on differences and similarities and then sorted into subcategories and categories describing the manifest content. In the last step, the categories were subsumed into themes, that is, an interpretation of the underlying pattern through the categories. The analysis resulted in three themes. Themes are threads of meaning that appear in one category after another (Woods and Catanzaro, 1988; Baxter, 1984).

### Ethical considerations

The participants were informed about the nature of the study and guaranteed confidentiality and an anonymous presentation of the findings. All signed a written informed consent before the interviews began. The first author informed the participants that they could withdraw from the study at any time without being questioned. Approval for conducting this study was given by the Regional Ethics Review Board (dnr. 2015-214-32M).

## Results

The analysis resulted in three themes: i) being supported by an encouraging contact, ii) being listen to and understood, and iii) being met with respect. The themes are presented below and illustrated with referenced quotes from the interviews.

### Being supported by an encouraging contact

Couples described a dignity encounter as having accessible PHC and being able to reach out to the healthcare personnel with a phone call or a face-to-face encounter when the ill partner needed help. Accessibility to care encouraged positive contact, which involved opportunities to ask the healthcare personnel questions directly. Couples described that when the care was accessible and they obtained advice and health counseling, a dignity encounter was promoted guidance in the right direction. They described that this facilitated their sense of being supported and able to manage the illness. Accessibility to care supported their feelings of being welcomed and understood, and when there was good accessibility, they felt as though the care they received fulfilled their needs.

… to get help and quick answers is fantastic … when the nurses go and talk directly to the GP while we are waiting on the telephone is fantastic and to receive help with our needs right away… situations like that promote being encountered with dignity. (Couple 3 interview)

A dignity encounter based on accessibility of care and beneficial contacts promoted a trusting relationship with healthcare personnel. Couples experienced that, with some healthcare personnel, it was easy to develop a good relationship, but with others, it was harder to reach out. They described situations when care was not accessible and that was when they were unable to contact or receive answers from healthcare personnel, they felt concerned, frustrated, and worried, which negatively affected their sense of being encountered with dignity. The couples described that it would be desirable to have a direct channel of communication to the healthcare personnel, as the current telephone system in PHC is difficult, and delays up to several hours are encountered in waiting for return calls. They described that it would be safer if access to healthcare personnel increased, as it promoted their sense of a dignity encounter.

… we have experienced certain problems to reach out to the healthcare personnel … the current telephone system in PHC is difficult to understand … which has made it difficult many times to contact the healthcare personnel when we have needed to … it contains so many steps … where you must press on different buttons … this is somewhat hard for people living with [a] visual impairment or [a] hearing problem … it would be more convenient if someone answered you directly when you’re calling: Hi, what can I help you with? (Couple 1 interview)

Couples described that a dignity encounter was when the GP called them by phone at home without any appointment. This allowed for fruitful conversations and beneficial contact as well as a faster way to get help with their needs. They described that a dignity encounter was involved being met in a peaceful environment and given sufficient time with the GP, the nurse, or other healthcare personnel. Couples claimed that dignity encounters should be planned without long waiting times and with a feeling of being welcomed in a peaceful atmosphere.

… to receive a dignity encounter from healthcare personnel, it ought to take place in a positive and relaxed atmosphere, where we can feel that we are welcomed and that we are not interfering with healthcare personnel. (Couple 5 interview)

### Being listen to and understood

Couples described a dignity encounter as when healthcare personnel confirmed them by listening to their stories. It facilitated a shared understanding concerning their experiences about the illness. When healthcare personnel showed that they understood the experiences related to illness, it was easier for patients to get close to the personnel. To be listened to enabled communication with the GP or the nurse and was described as a dignity encounter. Couples experienced healthcare personnel as attentive when they introduced themselves, made eye contact, and used human touch. A dignity encounter was also when healthcare personnel asked questions regarding their concerns and in what ways they could assist them with help. They described feeling that at such times healthcare personnel were caring for their health problems.

… a dignity encounter is to be seen by the one who receives you, and it is also extremely important to be listened to. Once you arrive (at the healthcare center), you do not want to feel as though you are disrupting something, since you are there for a reason, to be respected for who you are, and that what you are there for is important and not to be ignored. (Couple 4 Interview 4)

The couples described that dignity encounters meant that they were seen as credible persons with a great need to be helped. They wanted to be taken seriously and their views to be considered. When they visited the PHC, they said it was important that their stories and experiences were met with trustworthiness and that the healthcare personnel believed them.

… a dignity encounter in PHC means that they (healthcare personnel) believe in what you are asking and telling them about. (Couple 1 interview)

… to sense that the encounter has been a dignity encounter or not … has to do with if you feel that the healthcare personnel believe you or not. (Couple 2 interview)

Couples described that they had experienced an undignified encounter when they were not seen as credible and did not receive help they requested. Situations such as these gave them the impression of being invisible to the healthcare personnel. The couple described this as making them sad, angry, and feeling that their dignity had been violated. Encounters that failed to promote human dignity contributed to decreased health and well-being and to a loss of confidence in the care.

… we have experienced times when an encounter hasn’t maintained our dignity… and that was when we were not taken seriously although we had problems regarding the illness … the (GP) didn’t believe a treatment was indicated … but it appears that he was wrong and we received no apology. (Couple 3 interview)

### Being met with respect

Couples described that to be met with respect for their dignity contributed to feelings of being satisfied with the care they received. They described that, when they experienced dignity encounters, a feeling of safer care was improved. Dignity encounters that made them satisfied were closely connected to healthcare personnel’s abilities to embrace them with hope and happiness. They were satisfied when the healthcare personnel cared for them in a devoted way. To be satisfied with an inner thankfulness for being encountered with dignity gave them an overall positive impression of the entire healthcare personnel working in the PHC.

… dignity encounters are to be seen, to be listened to, to be acknowledged, and to be respected, and when that happens, you feel satisfied … in situations like that, the healthcare personnel show that they are professionals. (Couple 4 interview)

Couples described situations when they were dissatisfied with the encounter with the healthcare personnel. When this was the case, they wished to avoid encountering those healthcare personnel who had insulted them. The couples sometimes addressed their disappointment directly to the healthcare personnel, who then changed their attitude and became more responsive to their patients’ needs. They described it as important to speak up for themselves when dissatisfaction was a fact.

Couples described that a dignity encounter had a positive impact on their health and well-being; a dignity encounter gave them feelings of being safe and calm. Healthcare personnel who encountered them with dignity influenced their sense of satisfaction regarding their care and made them think of other things besides the illness. Couples were satisfied when the healthcare personnel had done “the little extra” for them; it made them happy. When they were satisfied with an encounter that had promoted their dignity, it etched in their minds good memories of received care and a sense of wanting to visit that PHC again.

**…** when you’re have a positive image of the healthcare center you visit, then it’s also easier to connect with the healthcare personnel, as you feel satisfied. (Couple 3 interview)

## Discussion

This study describes people with long-term illnesses and their close relatives’ experiences of dignity encounters within PHC. The findings show that couples experienced a dignity encounter when they were supported by an encouraging contact through accessibility to healthcare personnel. Accessibility facilitated beneficial contact with the healthcare personnel. Being listen to and understood was also important for experiencing a dignity encounter. Further, couples described a dignity encounter when they were met with respect which resulted in satisfaction with the care received at the PHC. A violated dignity based on nonchalance and ignorance led to feelings of being invisible in the encounter with healthcare personnel. In contrast, couples described that dignity encounters supported their health and well-being. According to Eriksson (1994) and Edlund (2002), a human being experiences her absolute dignity when feelings of being a presence and existing remain in the encounter with another. To experience dignity as a patient is fundamental and of great importance for every human health process. In this study, couples described that being satisfied increased their health and well-being and gave them feelings of happiness and hopefulness.

The findings show that couples experienced a dignity encounter when they were supported by an encouraging contact. Dignity encounters contributed to feelings of being welcomed and to being understood, and to feeling that they were being cared for. According to Barclay (2016), dignity is of particular importance in healthcare, as patients’ failing bodies, vulnerability, and loss of control over the healthcare environment negatively impact their ability to uphold values central in their lives. To be encountered by healthcare personnel with dignity and compassion is important, as it sends out signals that each person has equal worth, which adds something crucial to good healthcare. Nåden and Eriksson (2004) described that there are important values that preserve the dignity of patients, and aspects of those are described as inviting patients to play a role in their care, showing them genuine interest in their health and how they are feeling, and ensuring that they receive the help they need. It is important to establish a culture where the patients feel that they can talk openly about their needs. The couples experienced a dignity encounter as being met with advice and guidance that helped them to know what to do relate to their needs. Rehnsfeldt *et al*. (2014) stated that dignity is upheld when people with healthcare needs are respected as individuals and their needs and wishes are reflected upon. Patients described that their dignity had been maintained when they were paid attention to in terms of being recognized and when their individual needs were taken into account and healthcare personnel attempted to ascertain how their needs should be met (Matiti and Trorey, 2008). According to Widäng and Fridlund (2003), patients experienced dignity encounters when they were seen as a whole person with unique needs. When a patient was seen holistically, as an entity with physical, psychological, social, and existential needs, there was an increased possibility for well-being to be achieved. Therefore, it is essential that healthcare personnel understand people with long-term illnesses and their close relatives’ requests feel welcomed to care and to meet their needs.

The couples in this study experienced dignity encounters when they were confirmed by the healthcare personnel with an approach of being seen and listened to. Nåden and Eriksson (2004) stated that it is important that patients are enhanced with experiences of being understood, listened to, and seen. This is connected with healthcare personnel’s responsibility and respect for the patient. When patients are confirmed, important values are taken into accounts that preserve their human dignity. By approaching the patient with respect for his or her dignity also means caring for relatives and their needs (Lindwall and von Post, 2014). The findings show that couples valued a dignity encounter when healthcare personnel engaged with them with attention and interest, which was described as experiences of being important. According to Edlund (2002), is dignity a feeling of being somebody and of being important to others. When someone regards what the other person has to say, he or she is given confirmation of being worthy and being encountered with dignity. Studies (Walsh and Kowanko, 2002; Widäng and Fridlund, 2003) have shown that patients in inpatient care described dignity encounters as a way of being seen as a person. Part of being acknowledged was related to the recognition of the patient as a living, thinking, and experiencing human being and not as an object.

The findings show that couples have experienced the opposite of dignity encounters when they were encountered with nonchalance and ignorance, which gave them the impression of being invisible to healthcare personnel. This was expressed as their dignity having been violated, and it influenced their health and well-being negatively. Jacobson (2009) stated that dignity encounters should be built upon acceptance, love, and recognition for the other person. If a person is insulted and his or her dignity is violated, it can instead be destructive and lead to a feeling of being worthless as a human being. Studies (Skär and Söderberg, 2012; Söderberg *et al*., 2012; Nygren Zotterman *et al*., 2016) have shown that violating a patient’s dignity is to deprive the patient of his or her rights to be completely human in their wholeness. Unfortunately, violating patients’ dignity is a common form of care suffering.

Our findings show that couples experienced dignity encounters when they were viewed with credibility and trustworthiness and were taken seriously. This gave them a feeling of being understood by healthcare personnel. According to Eriksson (1994), human dignity is maintained in healthcare encounters when credibility is taken into account. Widäng and Fridlund (2003) stated that patients described that dignity encounters are related to how healthcare personnel greet them. They referred to dignity as being comprised of the sense of being seen as trustworthy, which meant that the healthcare personnel did not make negative statements that could lead to suffering. In this study, couples were sometimes encountered with mistrust in situations where a dignity encounter failed. According to Söderberg (1999), when patients are not believed and seen as credible, this could affect their dignity. Eriksson (1994) stated that if human beings are met with mistrust rather than credibility, it can cause suffering for the ones who are exposed and a feeling of not existing to the other person. Edlund (2002) describes that credibility and trustworthiness strengthen human dignity, while mistrust, disbelief, and being viewed as not believable instead lead to a violated dignity. According to Söderberg (1999), it is important to encounter people with illnesses with respect for their human dignity, as it is the foundation of all care.

The findings of this study show that the couples described the importance of dignity encounters as a way of being met with respect which contributed to their feelings of being cared for, secure, hopeful, and happy. This is consistent with Baillie (2009), who found that the meaning of patient dignity is related to feelings of being safe, happy, relaxed, and not worried, which can encourage their sense of well-being and feeling satisfied with their healthcare experience. According to Haddock (1996), when patient dignity is maintained in healthcare encounters, it is described as health promoting: the patient’s health could be re-examined and care could be facilitated so that the patient could manage his or her situation in a better way. The couples in this study described a dignity encounter as feeling safe and confident about the care they received within the PHC. Dignity encounters facilitated safer care, correct treatment, and positive opinions of the healthcare personnel. Similar to Beach *et al*. (2005), patients who were encountered with dignity in the healthcare setting also reported a higher satisfaction with their care, and this could also lead to a better adherence to treatments. Therefore, it is important to regard the patient and his or her close relatives’ experiences of dignity encounters.

### Limitations

Interviews with couples who had experienced encounters with healthcare personnel within PHC were used. When planning the interviews in pairs, it was important to reflect on ethical implications in order to promote a pleasant atmosphere for the participants to freely tell about their experiences. It was further important to make the participants feel confident during their participation and that the openness and intimacy that characterizes many interviews made the participant share their experiences. A joint approach may create tension between partners of the couple and harm the quality of the relationship (Zarhin, 2018). However, the authors were aware of the potential risk that the couple relationships could be ethically affected during the interviews. This type of interviews was assessed not affect the results because the couples had a shared experience of dignity in encounters in healthcare. The size of the interview group was relatively small, as the number of couples was five. This was, however, considered to be large enough to provide richness in the interviews, as the couples varied in age, diagnoses, and experiences of healthcare encounters. The participants were also very verbal and talked freely about their experiences of dignity encounters in healthcare. According to Sandelowski (1995), it is important that the sample size is large enough to reach variation in the participants’ experiences of the phenomenon yet small enough to permit a deep analysis of the data. At the same time, a small sample size can be a limitation if the participants contributed to so-called “elite bias” which reduces the generalizability of the results. However, our findings cannot be generalized, and this is not the goal of qualitative research, but the findings can be transferred to similar situations if they can be recontextualized to the actual context (cf. Polit and Beck, 2021).

The data collection method with couples is somewhat different from individual interviews. Interviews with couples have similarities with focus groups interviews, which involve a collective activity in which the participants aim to reflect on common experiences (cf. Kitzinger, 2005). Methodological considerations were careful regarding the data collection method and why interviews were not conducted with everyone separately. Our interest was to interview couples engaging in the interaction, and thus we concentrated on their common experiences, which were of interest (cf. Morgan and Spanish, 1984). Therefore, this data collection method suited our purposes.

## Conclusions

This study shows that dignity encounters, as experienced by people with long-term illnesses and their close relatives, involve being supported by an encouraging contact, being listen to and understood throughout the care process, and being met with respect by healthcare personnel. The couples experienced that a dignity encounter was to have accessibility to care that stimulated beneficial contact with healthcare personnel within the PHC, as they were often in need of their help. To receive a dignity encounter based on accessibility to healthcare personnel facilitated the couple’s experiences with support and guidance and in developing a trusting relationship. The couples described their experiences of being encountered with dignity when they were confirmed by healthcare personnel and seen by them as individuals, and when they were listened to. Furthermore, dignity encounters promoted their feelings of being satisfied with the care they received. To feel satisfied as because the encounter promoted their dignity had a significant positive impact on their health and well-being. This study shows that, for people with long-term illnesses and their close relatives, dignity encounters within PHC empower them for better health and well-being. Dignity encounters are, therefore, more than being treated well, it means to be strengthened in the relationship with healthcare personnel and be given opportunities to take part in decision-making. Based on this knowledge, healthcare personnel need to encourage and preserve dignity in their meetings with people with long-term illnesses and their close relatives, as doing so it can enable patients a sense of being understood and feeling supported in their needs. Dignity encounters can thereby improve the experiences of quality in healthcare encounters. Based on these results, further studies are needed to examine how healthcare professionals can develop their knowledge about dignity encounters.
